# If you organize, they will join

**DOI:** 10.1186/2045-3701-1-2

**Published:** 2011-01-18

**Authors:** Kuan-Teh Jeang

**Affiliations:** 1 The National Institutes of Health, Bethesda, MD, USA

## Abstract

This Editorial briefly introduces the Society of Chinese Bioscientists in America (SCBA) to readers of *Cell & Bioscience*.

## Introduction

There was a popular 1989 American movie that had a theme "if you build it, they will come" (Field of Dreams). When I saw that movie, I understood that it meant if you wish to do something good, don't wait for the approbations of others. Start, and if it is a good idea and you can show commitment, others will join you. Twenty-six years ago, three scientists (Horace Loh, CC Wang and Tommy Cheng) decided that it was time to organize a Society of Chinese Bioscientists in America (SCBA; http://www.scbasociety.org). Today, the SCBA is constituted by more than 2,000 bioscience principal investigators and younger scientists who are located primarily in North America and Asia. Loh, Wang and Cheng organized, and colleagues came and joined.

## A timely organization for the 21^st ^century

You shouldn't let the name fool you. You don't have to be based in North America to join. You don't even have to be ethnically Chinese to join the SCBA. Nationality is no more a requirement for you to join the SCBA than it is for you to be an American in order to join the American Society for Biochemistry and Molecular Biology (ASBMB) or the American Society for Microbiology (ASM). If you are interested in bioscience, the SCBA, like many international organizations, is interested in inviting you to join. Why should you join the SCBA? In an Editorial published in *Science *in 1993, Philip Abelson wrote "If the present dynamic pattern of growth continues, the future economic giants of the world will be located in the Western Pacific..." [1]. This year the size of the Chinese economy has grown to overtake that of Japan, easing it into second place only behind the United States. Moreover, statistics from the US National Science Foundation (NSF) showed that between 1995 and 2005, the output of worldwide science and engineering journal articles grew at an average annual rate of 2.3 percent; the US growth rate was 0.6 percent, while the greatest increase in annual article productivity came from Asia at an annual rate of 6.6 percent [2]. Indeed, in its November 2009 Global Research Report, Thomson Reuters (the publisher of ScienceWatch and Science Citation Index) stated that "by the measure of annual (scientific) output, China surpassed Japan, the UK and Germany in 2006 and now stands second only to the USA" [3]. As we enter the 21^st ^century, science in Asia is poised for further accelerated growth, and a professional society like the SCBA is arguably very well-positioned to promote the interests of the Western and the Asian scientific communities.

## The aims and activities of SCBA

The SCBA is a non-profit organization that has five objectives: 1) promote research in biosciences, 2) encourage advancement of biological and medical knowledge, 3) improve the qualifications and career opportunities of its members, 4) facilitate professional contact and interaction amongst bioscientists, and 5) build a spirit of fraternity and international cooperation in science. Initially established for bioscientists in the USA, the reach of the SCBA is now worldwide. On a biennial basis, the society organizes a large international meeting attended by 1,000 to 2,000 bioscientists. Our next meeting (the 13^th ^biennial SCBA International Conference) will be held on July 25-29, 2011 in Guangzhou, China http://www.scbameeting2011.org. This month, the SCBA is launching its first official journal *Cell & Bioscience *http://www.cellandbioscience.com. The journal is the long awaited fruition of the society's journal planning committee chaired by Chuxia Deng and CC Wang. With the leadership of Yun-Bo Shi, the editor-in-chief, *Cell & Bioscience *will publish high-impact research of broad general interest. The SCBA aims to be active and relevant. We welcome your interest and participation.
